# Chiral nanomaterials for tumor therapy: autophagy, apoptosis, and photothermal ablation

**DOI:** 10.1186/s12951-021-00965-7

**Published:** 2021-07-22

**Authors:** Zaihui Peng, Long Yuan, Juncheng XuHong, Hao Tian, Yi Zhang, Jun Deng, Xiaowei Qi

**Affiliations:** 1 Department of Breast Surgery, Southwest Hospital, Army Medical University, Chongqing, 400038 China; 2 Institute of Burn Research, Southwest Hospital, State Key Lab of Trauma, Burn and Combined Injury, Army Medical University, Chongqing, 400038 China

**Keywords:** Chirality, Nanomedicine, Biomaterials, Tumor therapy

## Abstract

Chirality is a fundamental characteristic of natural molecules and a crucial factor in the biochemical reactions of living cells and organisms. Recently, researchers have successfully introduced chiral molecules to the surfaces of nanomaterials, creating chiral nanomaterials that exhibit an upscaling of chiral behavior from the molecular scale to the nanoscale. These chiral nanomaterials can selectively induce autophagy, apoptosis, and photothermal ablation in tumor cells based on their chirality, making them promising for application in anti-tumor therapy. However, these interesting and important phenomena have hitherto received little attention. Accordingly, we herein present a review of recent research progress in the field of chiral nanomaterials for tumor therapy along with brief looks at the mechanistic details of their actions. Finally, the current challenges and future perspectives of chiral nanomaterials in terms of maximizing their potential in tumor therapy are discussed. Thus, this review provides a helpful introduction to the design of chiral nanomaterials and will hopefully highlight the importance of chirality in tumor therapy.

**Figure:**
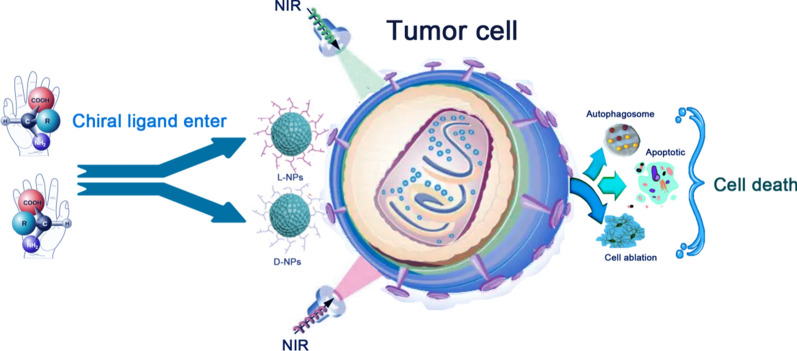

## Introduction

Chirality is a crucial property of natural molecules, chiral isomers have specific and selective effects on biological systems [1], which has extraordinary significance for a variety of biological events, including cell metabolism, cell fate, and even the evolution of organisms [2, 3]. For instance, in Eukarya, d-nucleotides, l-amino acids and l-phospholipids are homochiral building blocks from which live organisms are formed [4–6], while for bacterial, d-amino acids, i.e., d-Ala and d-Glu, present in the peptidoglycan on bacterial cell wall that act to provide resistance to most known proteases. Nanoparticles (NPs) have unique size and surface properties, which can enhance or expand their chiral effects [7, 8]. The introduction of molecular chirality into the surface of nanomaterials provides a new type of biological material—chiral nanomaterials. This new type of nanoscale chiral materials realizes a scale-leap for chiral signals from the molecular scale to the nanoscale, and exhibits superior properties to those of ordinary achiral nanomaterials [8, 9].

**Fig. 1:**
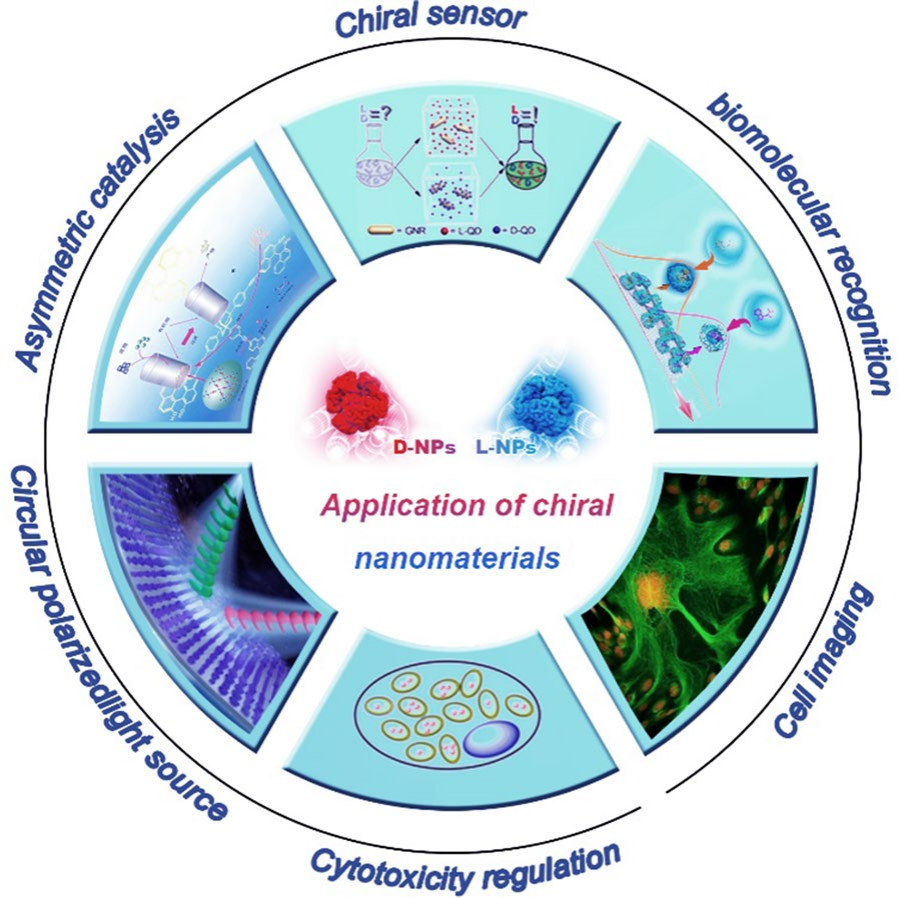
Some current applications of chiral nanomaterials

Related studies have found that the pharmacological activities of different enantiomers of chiral drugs sometimes show no obvious differences, but sometimes they may cause different or even opposite therapeutic effects. For example, in the antiarrhythmic effect of propafenone, its two isomers have similar pharmacological activities [10]. However, for the two enantiomers of propoxyphene, l-propoxyphene is a cough suppressant and d-propoxyphene is an analgesic. The two exhibit completely different pharmacological activities [11]. d-/l-dopamine is a drug used for the treatment of Parkinson's disease, but l-dopamine is therapeutically active, while d-dopamine can cause harm if it accumulates in the body [12]. Furthermore, the R-enantiomer of thalidomide has a therapeutic effect, while the S-enantiomer has a strong teratogenic effect [13]. The fascinating functions of chiral materials inspires tremendous amounts of research on developing functional chiral medicine [14]. For instance, chiral noble-metal NPs and quantum dots (QDs) that exhibit enantioselective catalytic properties and wide substrate generality have been reported [15, 16]. Furthermore, the interactions between nanomaterials and biological systems and their influence on phenomena such as cellular uptake and elimination have been widely studied as a means to further the application of chiral nanomaterials in biomedicine and bioengineering and to ensure their safety [17]. Moreover, chirality-based optical sensing and therapy strategies are becoming increasingly common [18–20]. The current applications of chiral nanomaterials are illustrated in Fig. 1.

Cancer is the greatest threat to human health and is becoming more serious because of factors such as environmental pollution and unhealthy lifestyle choices. Therapeutic strategies for clinical cancer treatment are dominated by traditional therapies involving surgery, chemotherapy, and/or radiotherapy. However, these traditional therapies are subject to several problematic factors [21–23] involving recurrence and secondary metastasis, poor tumor targeting, drug resistance, and radiation tolerance. Thus, strategies for enhancing the therapeutic effects and overcoming the problems of traditional therapies have been developed [24–27]. One such promising strategy is the use of chiral nanomedicine.

Many chiral nanomaterials have been fabricated for anti-tumor therapy. These materials can induce autophagy and apoptosis of tumor cells with high specificity and efficiency by virtue of the unique size and surface properties of NPs and the unique selectivity of the organism's chiral enantiomers [7, 8]. For example, chiral glutathione (GSH)-modified cadmium telluride (CdTe) QDs have been demonstrated to produce a large number of autophagic vacuoles in tumor cells and activate autophagy in a chirality-dependent way, where L-GSH-QDs induce more intense autophagy than D-GSH-QDs [28]. Furthermore, Du et al. modified the surfaces of NPs with L/D phosphotyrosine to synthesize chiral NPs (NP@-L/D-PYs). The alkaline phosphatase on the surface of cancer cells dephosphorylates the NP@-D-PYs, enabling them to adhere strongly to cancer cells, where they can activate exogenous cell death pathways and induce apoptosis. Interestingly, the NP@-L-PYs have almost no effect on cancer cells.

After the introduction of chiral amino acid ligands, a large number of amine groups and carboxyl groups are presented on the surfaces of the NPs, imparting excellent water solubility and suitable chemically reactive groups [29]. The chemically reactive groups attached to their surfaces being able to adopt a range of structural configurations, thus allowing electronic structures with different energy levels. This enhances the possibility for the recombination of electrons and holes captured by the surface states [30], which increases the fluorescence quantum yield of chiral NPs, which in turn makes them have excellent photothermal properties [31, 32]. Make chiral nanomaterials have broad application prospects in photothermal tumor treatment. For example, Li et al. used chiral cysteine to reduce MoO_3_, obtaining a dual visible- and near-infrared (NIR)-light-active nanomaterial (L/D-Cys-MO_3-X_ NPs) with strong chiral effects. After being endocytosed by tumor cells, the NPs generate temperatures in the range 40–50 °C upon absorbing circularly polarized light (CPL), thereby inducing cell death by denaturing intracellular proteins and rupturing cell membranes. L/D-Cys-MO_3-X_ NPs exhibit a higher photothermal conversion efficiency and tumor-cell-killing effect than traditional hyperthermic molybdenum oxide [33].

A considerable number of studies on the application of chiral ligands into nanomaterials in cancer therapy have been reported. Nevertheless, many questions remain to be answered. For example, do D or L chiral nanomaterials have better antitumor effects, and what are the mechanisms of their actions? Clearly, an informed summary of the biological functions and mechanisms of chirality in tumor therapy is needed. Accordingly, in this review, we consider the autophagy, apoptosis, and photothermal ablation of the three important types of tumor cells death as induced by chiral nanomaterials in separate sections. Then, the potential mechanisms by which chiral nanomaterials regulate tumor cells is briefly introduced. Finally, the future perspectives of chiral nanomaterials in terms of challenges currently faced in the field and some strategies to overcome them are presented. Overall, we hope to provide a theoretical basis and reference source for the development and application of new chiral nanomaterials in tumor therapy.

## Chiral nanomaterials that induce autophagy in tumor cells

Autophagy is a lysosome-based catabolic process that maintains homeostasis and the defense capabilities of cells by removing damaged and/or redundant proteins and organelles. Usually, after autophagy, endogenous and foreign substances are held in vesicles until they are finally degraded by lysosomes [34]. A series of studies have shown that autophagy has a close and complex relationship with the development and progression of tumors. For instance, autophagy can enhance the tolerance of tumor cells to tissue hypoxia, acidosis, and other adverse environments. Conversely, autophagy can eliminate damaged proteins and organelles to avoid genome damage, thereby suppressing the development of tumors. It is currently believed that during tumorigenesis, autophagy mainly inhibits the development and progression of tumor cells [35]. Thus, controlling the autophagy level of tumor cells may provide a promising strategy for the effective treatment of malignant tumors [36, 37], especially that of autophagy-deficient cancers, such breast, ovarian, and prostate cancers [38]. For instance, adjusting autophagy can change the sensitivity of triple-negative breast cancer cells to radiotherapy and reduce their resistance to chemotherapeutic drugs [39].

The features of chiral NPs, such as composition, size, shape, and surface functionalization, can regulate autophagy by inducing intracellular oxidative stress or changing the expression of autophagy-related genes/proteins [40–42]. Furthermore, recent related research has revealed that NPs as foreign materials in cells can influence autophagy [43].

**Fig. 2:**
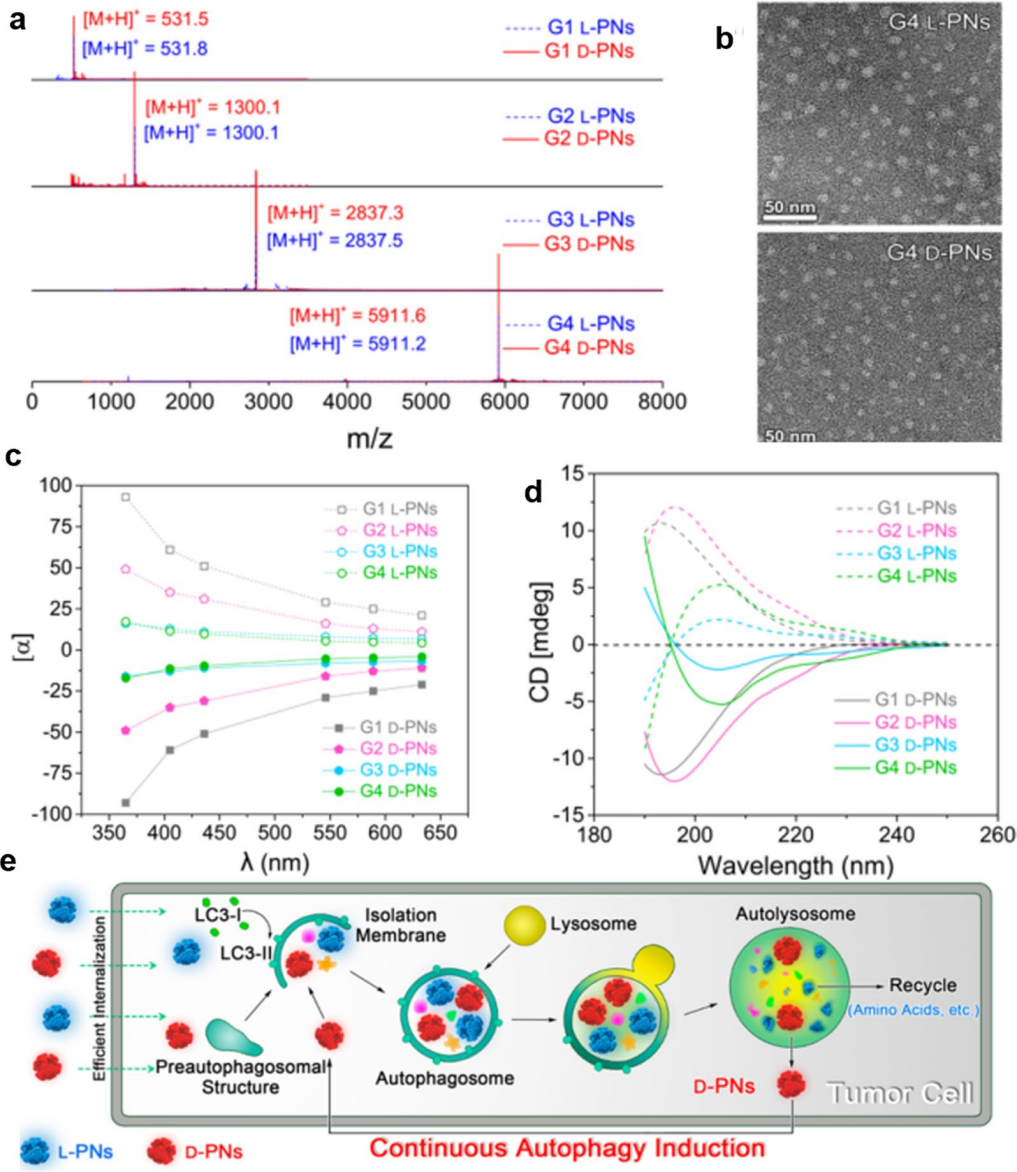
Characterization data for chiral L/D-PN protein nanomimics and a schematic of their effect upon autophagy processes and mechanisms. **a** Mass spectra; **b** transmission electron microscopy images; **c** optical rotatory dispersion curves; and **d** circular dichroism spectra. **e** Schematic of the influence of L/D-PNs upon autophagy processes. Reprinted with permission from ref. [51]. Copyright (2017) American Chemical Society

The chirality-dependences of cytotoxicity [44], cell differentiation [45, 46], cellular uptake [47], cell adhesion [48], and protein adsorption [49, 50] are widely accepted. Accordingly, NPs bearing surface-anchored chiral molecules have been widely demonstrated to modulate autophagy in tumor cells. For instance, in 2017, Hu’s group used natural chiral lysine to synthesize a chiral polymer with a molecular weight of ~ 5911 Da (Fig. 2a), that forms as unique L/D peptide dendrimers (L/D-PNs) with about 8 nm (Fig. 2b). In addition, the stereochemical properties of homochiral PNs were studied using optical rotation research (Fig. 2c, d). The L/D-PNs quickly activate the autophagy of tumor cells, inducing the formation of autophagosomes and autolysosomes (Fig. 2e). Moreover, this autophagy induction shows chirality dependence, with that by the D-type dendrimers being stronger than that by the L-type [51].

**Fig. 3:**
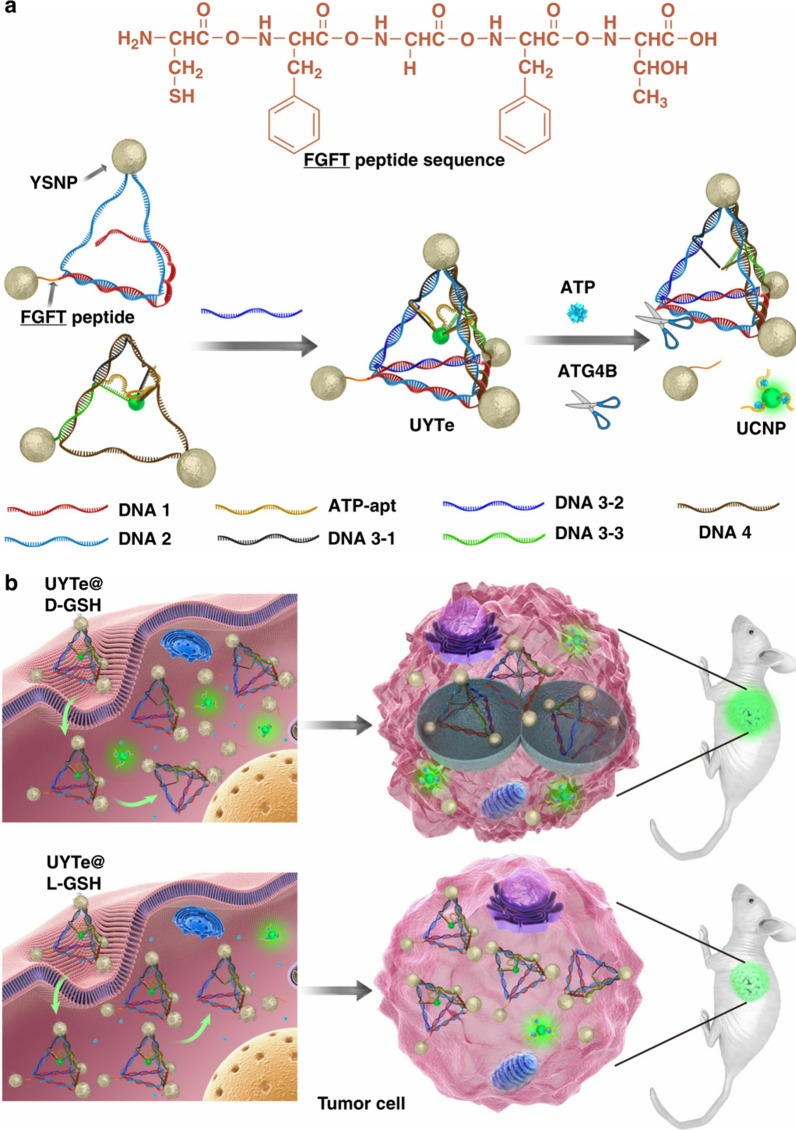
Schematic of a chiral nanodevice for autophagy induction and observation. **a** self-assembly of UCNP-centered yolk-shell tetrahedrons, and **b** their use to detect autophagy and ATP. Reprinted with permission from ref. [52]. Copyright (2018) Springer Nature

In 2018, Sun and coworkers used DNA self-assembly technology to assemble gold NPs (AuNPs) with core–shell structures into a chiral tetrahedral structure. One of the DNA ends was modified with a polypeptide chain that could be hydrolyzed using the autophagy marker ATG4b, and up-conversion NPs (UCNPs) were further assembled in the tetrahedron using ATP aptamers. Modification of d/l-glutathione on the surface of the assembly enabled the tetrahedron to present tunable circular dichroism (CD). When tetrahedrons with embedded UCNPs are cultured with cancer cells, the d-glutathione-modified tetrahedron structure causes the cells to produce a strong autophagy response, and this autophagy process can be monitored in real time through CD and up-conversion luminescence measurement, as illustrated in Fig. 3 [52].

Wang et al. reported the fabrication of D- and L-cysteine-modified Cu_2-x_S nanocrystals (NCs) using a sacrifice template-ligand exchange strategy. Their interactions with cells and ability to induce tumor cell autophagy as well as their photothermal ablation properties were investigated. The authors reported that the NCs induce the production of large amounts of reactive oxygen species (ROS) in tumor cells, which promotes cellular autophagy. Furthermore, the cell ablation is improved by the photothermal effects of the Cu_2-x_S NCs, and the autophagy activation was found to be chirality dependent, with D-Cu_2-x_S NCs causing more extensive autophagy than L-Cu_2-x_S NCs [53].

**Fig. 4:**
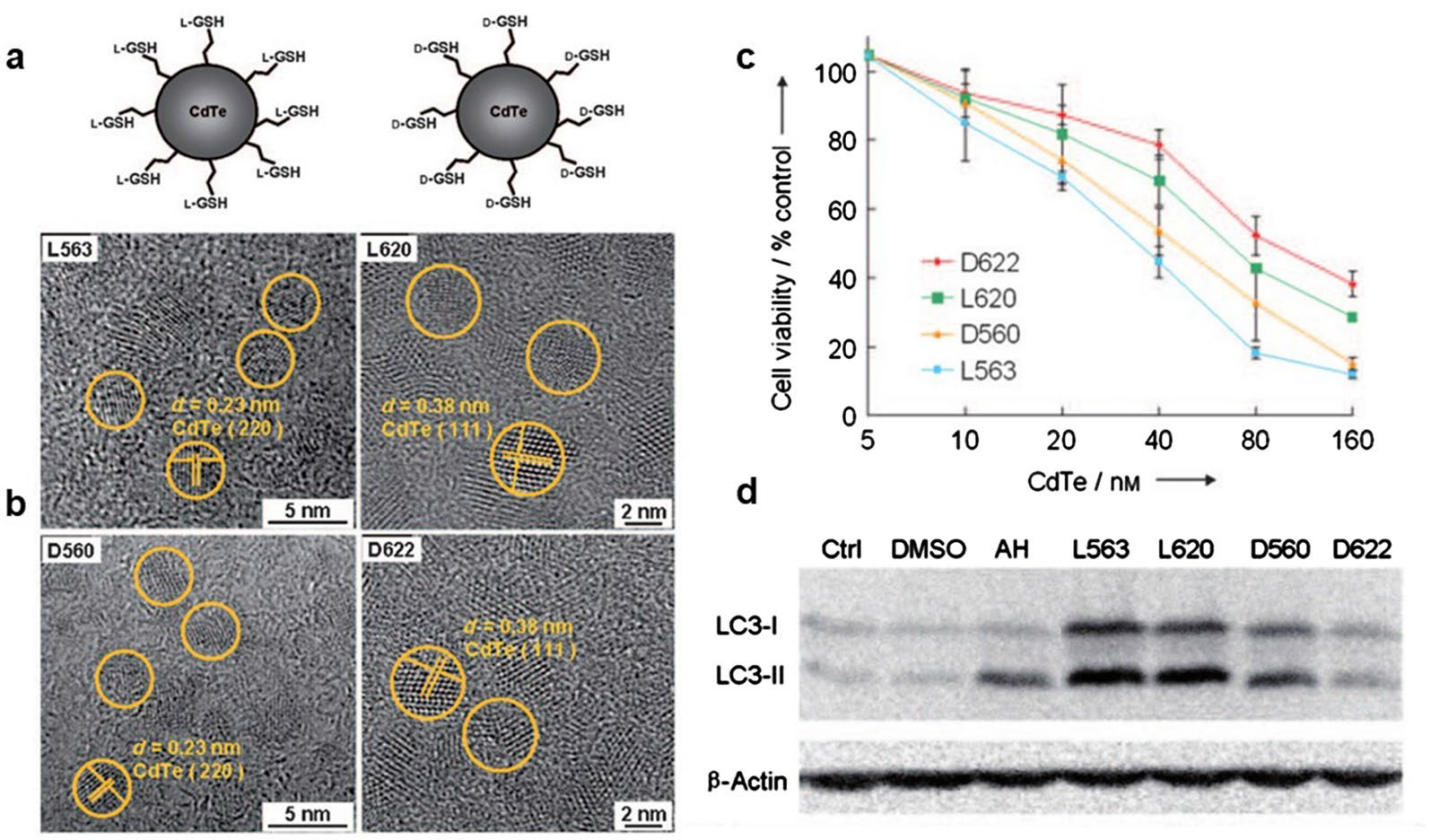
**a** Schematic structures of L- (left) and D-GSH-QDs (right). **b** High-resolution transmission electron microscopy images of D- and L-GSH-QDs. The circles indicate the spacings of the CdTe (220) or (111) lattices. **c** Chirality- and concentration-dependent cytotoxicities of the QDs. **d** L-GSH-QDs induce autophagy more effectively than D-GSH-QDs (40 nm). Reprinted with permission from ref. [28]. Copyright 2011 WILEY‐VCH Verlag GmbH & Co. KGaA, Weinheim

Interestingly, and as is inconsistent with the results of the above study, Li et al. attached chiral GSH to the surface of CdTe QDs to obtain chiral QDs (L/D-GSH-QDs), as illustrated in Fig. 4a. Transmission electron microscopy (TEM) indicated that all QDs are approximately spherical in shape. The lattice spacing of the (220) plane for D560 and L563 QDs showed a distance of 0.23 nm, and of the (111) plane for D622 and L620 QDs a distance of 0.38 nm (Fig. 4b). The chiral QDs were co-cultured with human liver cancer cells, revealing that both L- and D-GSH-QDs exhibit dose-dependent cytotoxicity and significantly increase the levels of autophagic vacuoles. However, the autophagy activation was found to be chirality-dependent, with L-GSH-QDs being the more effective (Fig. 4c, d) [28].

**Fig. 5:**
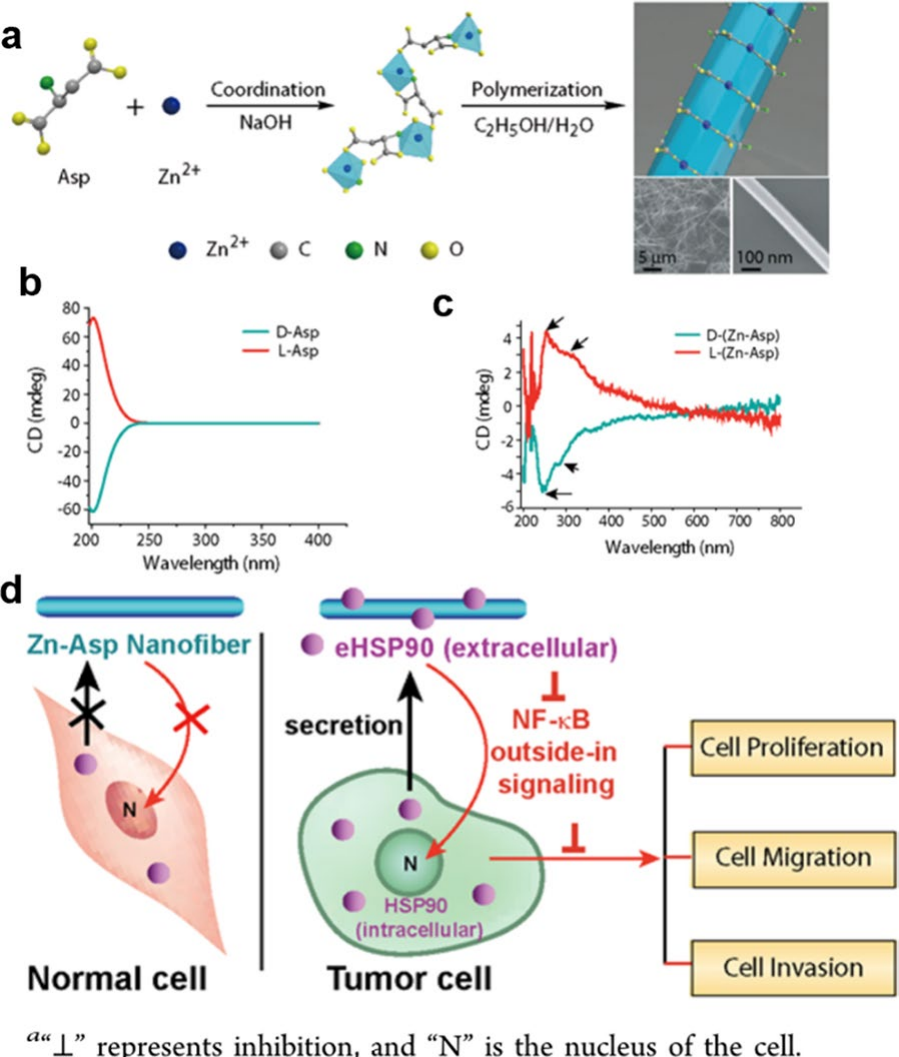
Schematics of the polymerization and autophagy regulation action of L/D-(Zn-ASP) NFs. **a** Coordinative polymerization and high-resolution scanning electron microscopy images of L/D-(Zn-ASP) NFs. CD spectra of **b** D-and L-Asp and **c** D- and L-(Zn-Asp) NFs, **d** How L/D-(Zn-ASP) NFs regulate autophagy. Reprinted with permission from ref. [54]. Copyright (2015) American Chemical Society

Xin et al. synthesized novel chiral zinc-aspartate nanofibers (L/D-(Zn-ASP) NFs) that were found to be over 30 μm in length and highly uniform with diameters < 100 nm by an interfacial polymerization reaction between L/D aspartate and zinc ions (Fig. 5a–c). The polymer retained in the extracellular environment can strongly interact with eHSP90 from the cancer cell, which induces decrease in the level of gelatinases, resulting in downregulation of nuclear factor-kappa B (NF-κB) signaling and formation of autophagy, finally inhibiting cancer cell proliferation, migration, and invasion (Fig. 5d) [54].

**Fig. 6:**
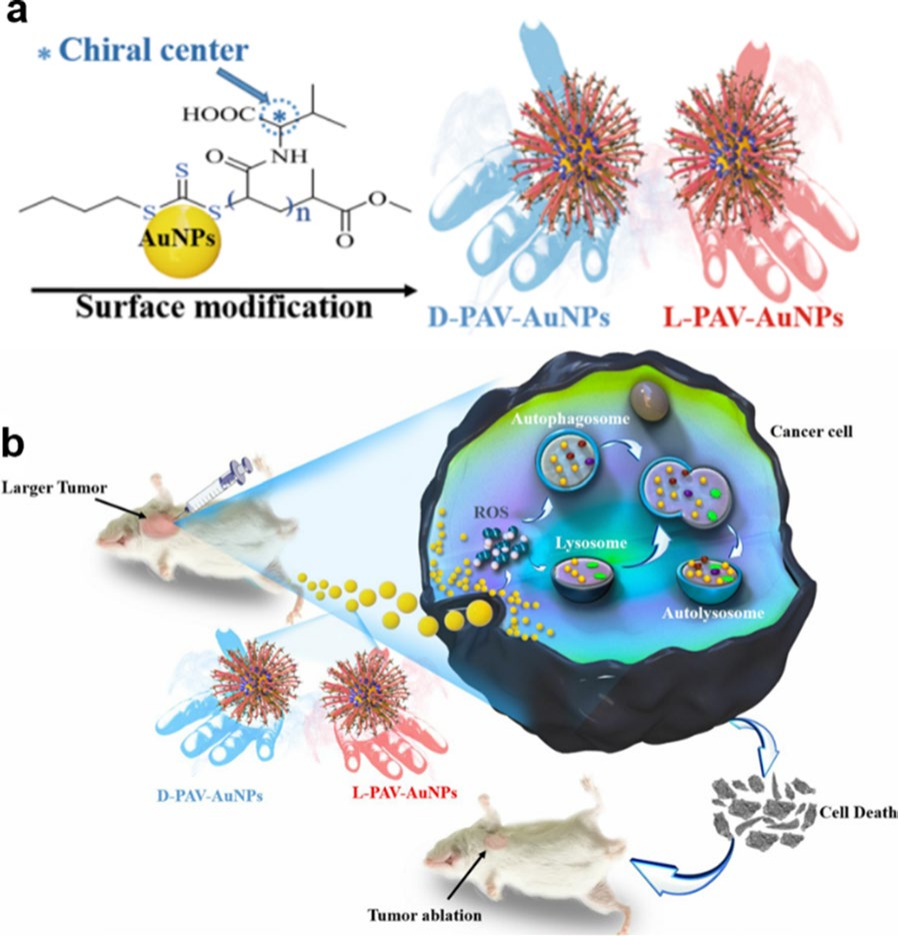
Schematics illustrating the synthesis of L-PAV-AuNPs, D-PAV-AuNPs, and L/D-PAV-AuNPs and the mechanism of their chirality-dependent autophagy activation. **a** Synthesis of L-PAV-AuNPs, D-PAV-AuNPs, and L/D-PAV-AuNPs and their conjugation to AuNP surfaces. **b** Mechanism of chirality-dependent autophagy activation by L-PAV-AuNPs, D-PAV-AuNPs, And L/D-PAV-AuNPs and its application in tumor therapy. Reprinted with permission from ref. [55]. Copyright (2018) BioMed Central

Research on chiral dependence in autophagy induced by nanomaterials is still at an early stage and there are many controversies. To explore this topic, our group synthesized AuNPs coated with the L, D, and racemic forms of chiral poly (acryloyl valine) to form L-PAV-AuNPs, D-PAV-AuNPs, and L/D-PAV-AuNPs, respectively (Fig. 6a). We found that L(D)-PAV-AuNPs induce ROS and lysosome function, thereby causing cells to undergo autophagy and cell death, in a chirality-dependent manner. The inhibitory effect of D-PAV-AuNPs was found to be stronger than that of the L form (Fig. 6b). Additionally, L(D)-PAV-AuNPs show very low toxicity to normal 3T3 fibroblasts and breast epithelial cells, have the ability to induce tumor cells with high selectivity, and thus have great potential for biological application [55].

The above studies show that chiral nanomaterials that induce autophagy in tumor cells do so in a chirality-dependent manner, with the dextrorotatory-polymer-modified nanomaterials having a stronger autophagy induction effect. This phenomenon may be due to the main way for NPs to enter cells is by micropinocytosis [47]. After L/D-NPs enter the cell, the L-phospholipid molecular layer on the surface of the cancer cell is selectively more inclined to combine with heteroenantiomeric polymers (D-NPs). As a result, regardless of the concentration of chiral NPs, D-NPs show greater absorption than L-NPs [52]. As D-NPs accumulate, more ATP will be produced by the reaction of NPs with biological macromolecules in the cell [4], and autophagy-related reactions depend on the expression of ATP [56, 57]. D-NPs accumulate more, and the interaction with cell surface receptors induces more ROS [55], ROS are necessary for inducing autophagy [58], and thus induce the autophagy of cancer cells to a greater extent. Secondly, the biological activities and metabolism of endogenous proteins are regulated by chirality-specific enzymes. Compared with L-NPs, D-NPs are not easily degraded by natural proteases and coenzymes in the cellular environment. It may be precisely because D-NPs are tolerant to biodegradation that they can more continuously and efficiently induce autophagy [33, 59]. In addition, the amino acid moiety of the chiral NPs could form hydrogen bond with the specific protein secreted by cancer cells, which might enhance the binding interaction [60], and the intensity of the hydrogen bonds between chiral NPs and specific protein can be different considering the different spatial arrangements of the functional groups (the uncoordinated carboxyl and amine group of chiral Asp) on the surface of the two kinds of NPs[61]. Therefore, the binding between D-/L-NPs and specific protein are not exactly the same, resulting in a slight difference in their autophagy ability toward cancer cells.

Amino acids have been widely used for studying the interaction between cells and chiral surface due to their versatility and biocompatibility [62], and amino type could influence the chiral effects on biological systems [63]. Interestingly, in the studies discussed above, Sun et al. [52] and Li et al. [28] both used glutathione for chiral NP fabrication, but their results for the chirality-dependence of autophagy induction in cancer cells are completely opposite. This may be because the former mainly benefits from the stronger uptake of D-type NP by cells, while the abundance of L-GSH and relative scarcity of D-GSH in biological systems may lead to differences in the shell degradation behavior of the QDs. GSH-coated QDs will more readily interact with GSH of the same enantiomer. This difference makes the thermodynamic stability of QDs L-chiral dependent.

These results provide insights for the development of new chiral NPs that induce autophagy in tumor cells in a chirality-dependent manner. However, it should be noted that the complexity and heterogeneity of biomolecules and cells mean that many questions have yet to be answered and ongoing research must be performed.

## Chiral nanomaterials that induce apoptosis in tumor cells

Apoptosis, also known as programmed cell death, is a physiological process involving multiple factors, including the immune response, gene regulation, and signal transduction [64]. Apoptosis plays a very important regulatory role in a variety of normal physiological processes. Thus, abnormal apoptosis can give rise to a range of pathological effects, such as tumors, autoimmune diseases, and neurodegenerative diseases [65]. Studies in recent years have confirmed that the unregulated growth of tumor cells is the result of inhibited tumor cell apoptosis. Therefore, apoptosis disorders are closely related to tumor occurrence, development, and regression [66]. Accordingly, several scholars believe that inducing apoptosis in tumor cells may become a viable means of treating cancer [67]. Furthermore, recent studies have demonstrated that NPs can activate caspase-family proteins as well as the expression of DNA-damage-repair proteins and pro-apoptotic gene P53, thus promoting cancer cell apoptosis [68–70].

The functionality of a biological system is entirely dependent on the physical structures of its components. This means that, in chirality-specific biological interactions, one enantiomer of a molecule can have an important biological function while the other may be inactive or even harmful. Thus, numerous researchers have applied chiral nanotechnology to induce apoptosis in cancer cells.

**Fig. 7:**
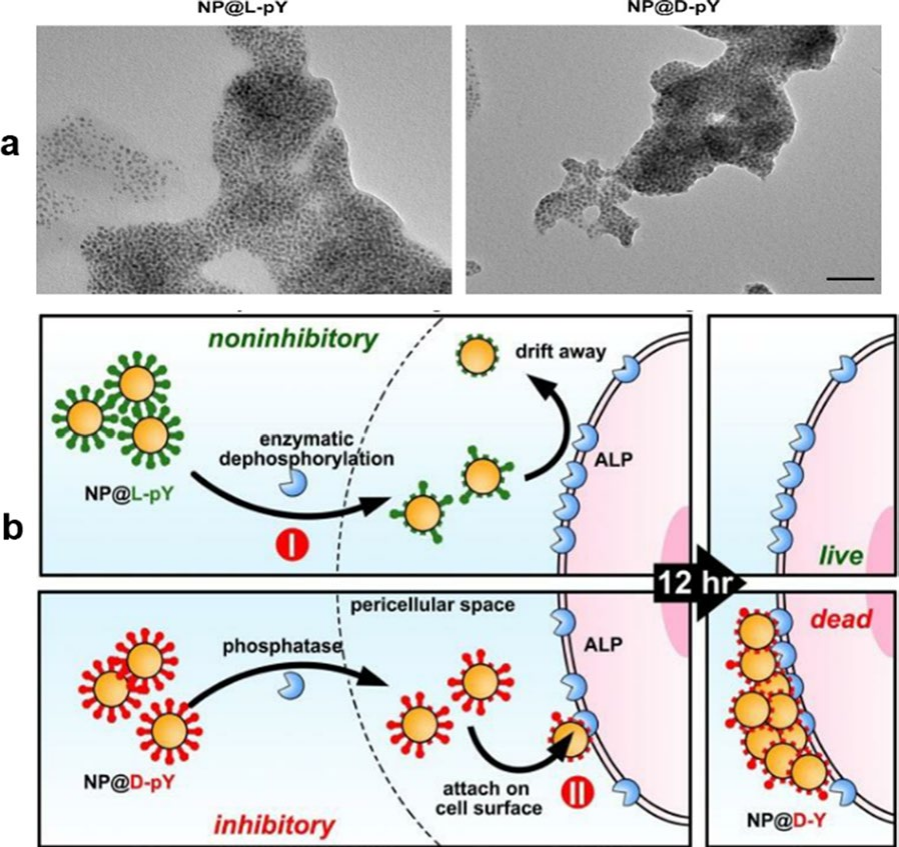
Chiral-NP-controlled reaction–diffusion leads to cell death. **a** Transmission electron microscopy images of NP@L-pYs (left), and NP@D-pYs (right). Scale bar = 50 nm. **b** Enzymes in the culture medium dephosphorylate the pY groups on the NP@L-pYs but not those on the NP@D-pYs. Then, the alkaline phosphatases on the cancer cell surfaces dephosphorylate most of the pYs remaining on the NP@D-pYs, causing them to adhere to the cells and evoke apoptosis. Reprinted with permission from ref. [71]. Copyright (2017) Wiley–VCH Verlag GmbH & Co. KGaA, Weinheim

Du et al. decorated iron oxide NPs with D/L-phosphotyrosine to form bioactive magnetic NPs (NP@D/L-pYs) with diameters of ~ 10 nm. These NPs cluster somewhat to form small aggregates with an average diameter of 500 nm (Fig. 7a). When cancer cells are co-cultured with the NPs, the alkaline phosphatase on the surface of the cancer cells dephosphorylates the pY moieties, allowing NP@-D-Y to strongly adhere to the cancer cells and activate exogenous cell death, causing apoptosis (Fig. 7b) [71].

**Fig. 8:**
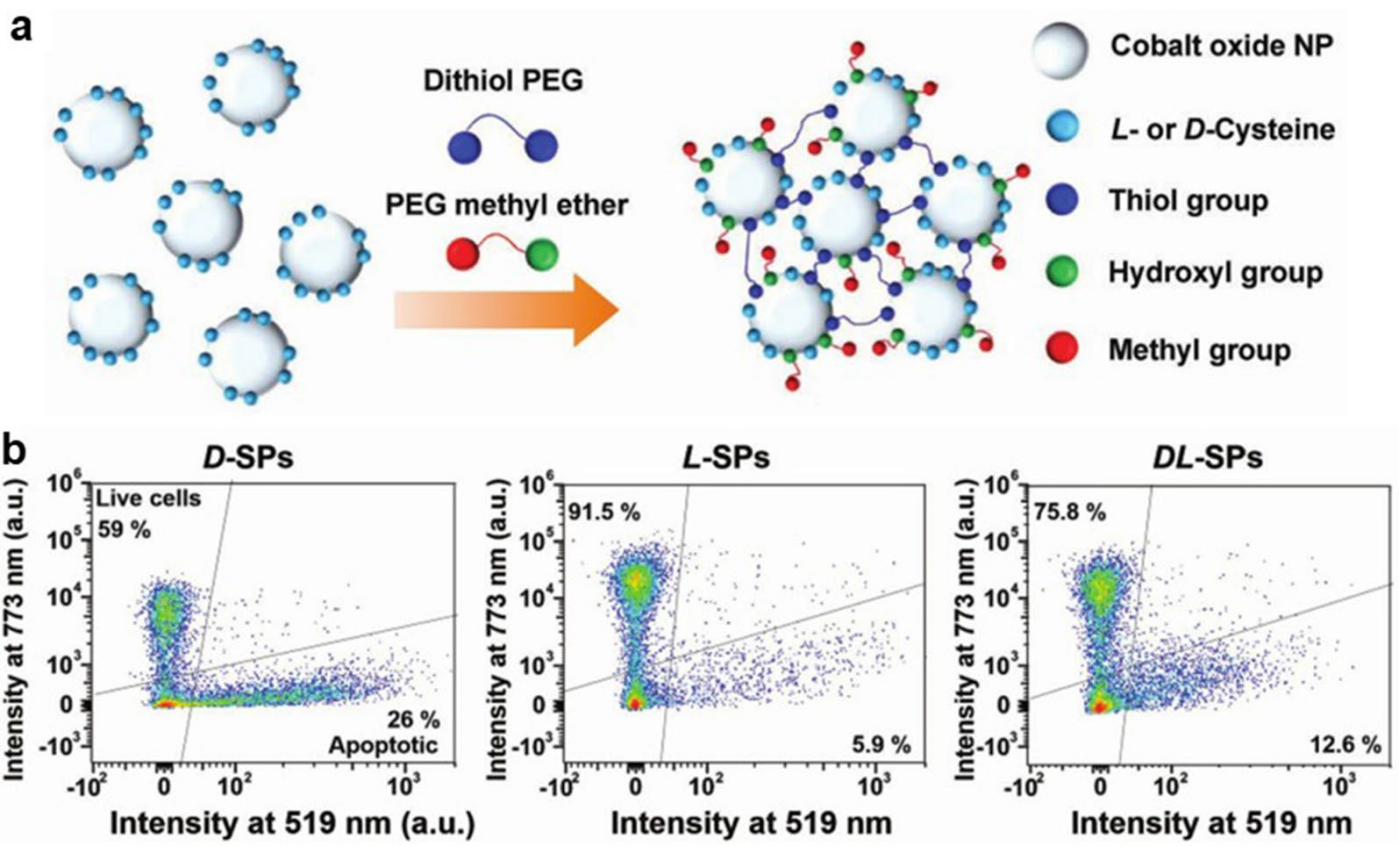
**a** Schematic showing chiral NPs self-assembling into SPs. **b** Mitochondria membrane potential-dependent fluorescent signals after HeLa cell treatment with D-, L-, and racemic SPs. Reprinted with permission from ref. [9]. Copyright (2019) WILEY–VCH Verlag GmbH & Co. KGaA, Weinheim

In 2019, Yeom et al. assembled chirality-engineered supraparticles (SPs) from small chiral CdTe NPs and modified their surfaces with chiral cysteine to synthesize ultrafine chiral SPs (L/D-SPs) (Fig. 8a). The ultrafine chiral particles damage the mitochondrial membranes of cancer cells, thereby causing apoptosis, in a chirality-dependent manner whereby d-SPs have a stronger apoptosis-inducing effect than L-SPs (Fig. 8b) [9][9].

**Fig. 9:**
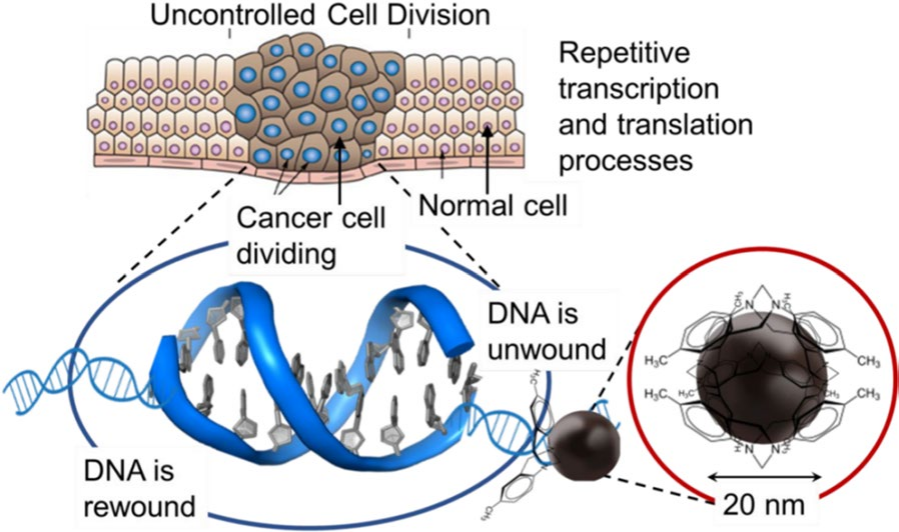
Schematic showing cancer cell apoptosis caused by the interaction between chiral carbon NPs and DNA. Reprinted with permission from ref. [72]. Copyright (2018) Wiley–VCH Verlag GmbH & Co. KGaA, Weinheim

Tripathi et al. imparted chirality in carbon nanoparticles by controlled tethering of chiral molecules, i.e. TrÖger’s base. It was found that chiral particles present groove-binding agents at the nanometer level, which can be positively enriching the cellular nucleus in MCF-7 breast cancer cells and give DNA enantiomer-specific recognition and help induce the apoptotic cascade of cancer cells. However, the negatively induced chiral carbon nanoparticles showed higher efficiency in inhibiting cell growth [72]. Schematic showing cancer cell apoptosis caused by the interaction between chiral carbon NPs and DNA (Fig. 9).

**Fig. 10:**
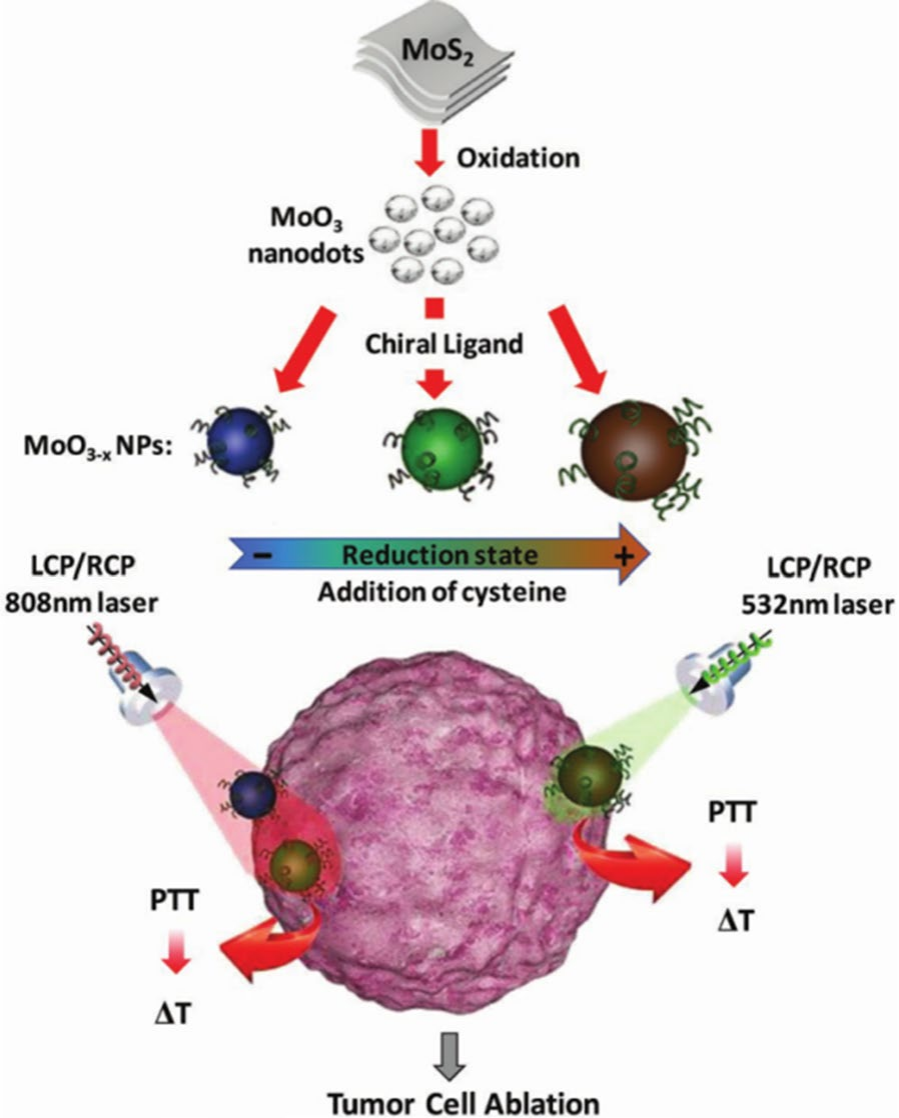
Schematic showing the fabrication of Cys-MoO_3-x_ NPs and their use in photothermal tumor ablation. Reprinted with permission from ref. [33]. Copyright (2018) WILEY–VCH Verlag GmbH & Co. KGaA, Weinheim

The above studies demonstrate that introducing chirality into a nanosystem can enhance its ability to evoke cancer cell apoptosis. In terms of underlying mechanism, the apoptosis-inducing effect of D-NPs is stronger than that of L-NPs due to the alkaline phosphatase secreted by cancer cells that dephosphorylates NP@L-pYs more rapidly than NP@D-pYs. The NP@L-pYs are mostly dephosphorylated before reaching the cancer cells. Therefore, there is no phosphate group and the NPs cannot bind to and kill the cell. However, the surface of NP@D-pYs is dephosphorylated at the cancer cells. This in-situ dephosphorization forms NP@D-Ys, which bind to cancer cells induces their apoptosis [71].

In addition, the D-chirality matches that of the L-phospholipid molecular layer on the surface of the cancer cell, leading to more efficient binding between them, and D-NPs are not easily degraded by natural proteases and coenzymes in the cellular environment, Both of these make D-NPs better absorbed by cancer cells and induce their apoptosis.Moreover, the efficacy of the negatively induced chiral carbon NPs in cancer treatment may due to the low intracellular PH value, the negatively induced chiral carbon NPs can more easily enter in nucleus by lipid-flipping thereby interacting with DNA in a grooved manner [72].

These results provide insights for the development of new chiral NPs that induce tumor cell apoptosis more effectively, thus realizing efficient targeted tumor therapy.

## Chiral nanomaterials for photothermal tumor therapy

Photothermal therapy is a cancer-treatment strategy that involves local heat generation in cancer cells upon light radiation [73]. This technology uses NIR light to irradiate tumor tissues as it exhibits strong tissue penetration. Photothermal conversion then increases the temperature of the tumor tissue, thereby damaging tumor cells through protein denaturation and membrane disruption [74, 75]. In recent years, several studies have demonstrated that the heat generated by nano-photothermal materials not only has the effect of directly killing tumor cells, it can also inhibit tumor metastasis [76–78]. In addition, nano-photothermal materials can also be used in bioimaging, and they may also be modified in such a way as to impart properties that may be beneficial to chemotherapy, radiotherapy, and immunotherapy, making them effective multifunctional diagnostic and therapeutic agents against tumors [79, 80]. Accordingly, the integration of diagnostic and therapeutic functions into single nanomaterials will realize the integration of nanomedicine in tumor-imaging diagnosis and treatment.

Combining chirality with specific selectivity for biological systems and NPs with special size and surface properties-chiral NPs, which have more significant photothermal properties, and can be better applied to photothermal treatment of tumors [81–83]. For instance, Chen et al. introduced L-cysteine to the surface of molybdenum (MoO_3_ − x) NPs with a large optical adsorption coefficient and successfully synthesized chiral molybdenum (Cys-MoO_3_-x) and applied them to squamous cell carcinoma (OSCC) cells and found that they had a visible light (VL) to NIR dual PTT effect, with tumor cell fatality rates up to 89% [82]. Antaris et al. modified (6,5) chiral SWCNTs with C18-PMH-mPEG to fabricate biocompatible SWCNTs. Chiral carbon nanotubes are highly biocompatible and therefore exhibit extended blood circulation and high tumor-uptake rates without losing their NIR photoluminescence (PL) properties. The SWCNTs were injected intravenously into a mouse tumor model and successfully used to heat the tumors photothermally to over 50 °C while simultaneously visualizing tumor accumulation through whole-animal NIR-PL imaging [84]. Zhao et al. prepared HER2 aptamers of SK-BR-3 breast cancer cells and used them in the preparation of chiral Ag@Au core–shell NP assemblies (Ag@Au CS NPs). The NPs were demonstrated to be stable, and they allowed sensitive and accurate detection of cancer cells that exhibit HER2 overexpression [85].

Yang et al. introduced N-isobutyryl-L(D)-cysteine enantiomers as stabilizers into HgCl2 and Na2S aqueous solutions as a means to synthesize chiral QDs (β-HgS QDs). An aqueous β-HgS QD solution was heated to 50 °C upon laser irradiation, and the photothermal conversion efficiency was found to be stable over several cold-thermal cycles [59]. Li et al. used chiral cysteine as a reducing agent to reduce MoO3, obtaining a dual visible-light- and NIR-active nanomaterial (L/D-Cys-MO3-X NPs) with a strong chiral effect (Fig. 10). After being endocytosed by tumor cells, the NP generate temperatures of 40–50 °C in tumor cells upon absorbing visible- or NIR-CPL, thereby inducing cell death by denaturing intracellular proteins and rupturing cell membranes. L/D-Cys-MO3-X NPs exhibit higher photothermal conversion efficiency and tumor cell killing effect than the traditional hyperthermic agent molybdenum oxide. Notably, these chiral NPs show chiral selectivity for incident light. When irradiated by left-CPL, L-Cys-MoO3-x has the highest efficiency for HeLa cell ablation in vitro, while the corresponding results are observed for D-Cys-MoO3-x and right-CPL [33]. Li Feng et al. synthesized chiral N-S-doped carbon dots in aqueous solution using cysteine, and their PL quantum yields were found to reach 41.26%. Interestingly, the chiroptical properties and fluorescence intensity of the N-S-doped carbon dots are pH dependent. Additionally, L-carbon dots effect chirality-dependent glycolysis enhancement in T24 bladder cancer cells [86]. These studies demonstrate that chiral ligands can dramatically improve the optical properties of compounds, enabling their application to photothermal therapy and optical imaging of tumor cells.

In terms of mechanism, the introduction of chiral ligands improves the biocompatibility of a nanomaterial, increasing its blood-circulation half-life and thus uptake by tumor cells. Furthermore, the introduction of chiral ligands can improve the cancer-cell bioaffinity of a nanomaterial, strengthen its resistance to peptides and proteins in the blood, and endow it with chiral optical activity, NIR absorption, fluorescence emission, and photothermal conversion. There is a big chirality difference between L(or)D-NPs and Circulating tumor DNA (ctDNA). The influence of steric hindrance makes D-NPs more likely to bind to B-helix ctDNA than L-NPs, which may also be one of the reasons [87].

Based on the above studies, we believe that chiral recognition will come to play an important role in photothermal therapy. Based on the chiral activities, NIR fluorescence, biocompatibilities, chiral-recognition potential, and stable and efficient photothermal conversion, chiral nanomaterials are promising candidate agents for photothermal therapy and/or NIR fluorescent probes.

## Conclusions and future perspectives

In summary, chiral nanomaterials can kill tumor cells and inhibit tumor growth by inducing cell apoptosis, autophagy, and photothermal ablation. The reasons as follows. Firstly, after the introduction of chiral amino acid ligands, a large number of amine groups and carboxyl groups are presented on the surfaces of the NPs, imparting excellent water solubility and suitable chemically reactive groups [29], which can greatly improve their biocompatible [51]; Secondly, Good endocytosis is the crucial first step for NPs accumulation in the cell. NPs as extracellular substance the main way to enter cells is micropinocytosis [47]. After enter the cell, the phospholipid layer on the surface of the cancer cell can combine with chiral NPs. Additionally, chiral NPs are intercalated between the G-C bases of circulating tumor DNA (ctDNA), thereby affecting the base stacking of ctDNA [88].

However, the autophagy, apoptosis, and photothermal ablation of tumor cells induced by chiral NPs appear to be D-chirality-dependent. In terms of potential mechanism: (1) due to the L-phospholipid molecular layer on the surface of the cancer cell is more likely to combine with heteroenantiomeric polymers (D-NPs), making D-NPs more easily absorbed and accumulated by cancer cells [52]. As D-NPs accumulate, more ATP will be produced by the reaction of NPs with biological macromolecules in the cell [4], and autophagy-related reactions depend on the expression of ATP [56, 57]. D-NPs accumulate more, and the interaction with cell surface receptors induces more ROS [55], ROS are necessary for inducing autophagy [58]. (2) The biological activities and metabolism of endogenous proteins are regulated by chirality-specific enzymes. Compared with L-NPs, D-NPs are not easily degraded by natural proteases and coenzymes in the cellular environment. It may be this biodegradation tolerance that allows them to continuously and more efficiently induce autophagy [33, 59]. (3) The alkaline phosphatase secreted by cancer cells dephosphorylates L-NPs faster than D-NPs. L-NPs are mostly dephosphorylated before reaching cancer cells. Therefore, without phosphate groups, L-NPs cannot bind to and kill cancer cells. However, the surface of D-NPs is dephosphorylated on cancer cells. This in situ dephosphorization method allows binding to cancer cells and inhibition of their growth [71]. (4) The intercalation binding between chiral-NP and G-C base of ctDNA is affected by steric hindrance, the binding ability of D-NPs and ctDNA is stronger than that for L-NPs, thereby inducing the death of cancer cells more efficiently [88]. Of course, there are exceptions. In the study of Li’s group, the abundance of L-GSH and relative scarcity of D-GSH in biological systems lead to differences in the shell degradation, and ultimately in the autophagy induced by L-GSH-QDs being stronger than that by D-GSH-QDs[28]。

More interestingly, in some of the above researches, chiral NPs can discriminate model tumor cells from normal cells. This may be due to upon contact with the complex biological systems, the proteins will be progressively and selectively adsorbed on the NPs surface, which was defined as “protein corona” [89, 90]. Chiral NPs can specifically bind to tumor cell-related proteins, but have low affinity with normal cells [4]. An example is the high-affinity binding of chiral NPs to heat shock protein (HSP90) [54], which plays an important role in tumor progression [91]. Chiral NPs can interact strongly with transferrin [4], which is highly expressed in cancer cells [92] and shows high-affinity [93], to achieve tumor targeting [4]. In addition, the looser cytomembrane structure [94] and larger surface viscosity [95] tumor cells are more easily to be invaded as compared with the normal ones.

The commonality of their underlying mechanisms presents the possibility of developing nanomaterials that induce both tumor autophagy and apoptosis. In addition, the optical properties can also be significantly improved by introducing chiral ligands, allowing application to photothermal therapy and optical imaging of tumor cells. Thus, the synthesis of chiral nanopolymers may realize the combination of autophagy induction, apoptosis induction, photothermal therapy, and bioimaging.

However, despite the substantial achievements in the application of chiral nanomaterials to the field of tumor treatment, there remain issues and problems to be addressed. Firstly, most of the inorganic NPs reported are cadmium and/or gold based [28, 55]. These materials are either intrinsically biotoxic or non-biodegradable and therefore suboptimal for investigating chiral effects on nano-bio interactions. Furthermore, these materials have side effects in biological applications. Thus, to better explore the application of chiral nanomaterials in biomedical, a promising strategy is to utilize non-toxic, without side effects and degradable raw materials. Secondly, although they exhibit prominent advantages in terms of blood circulation and tumor accumulation, chiral nanomaterials are always subject to two major pharmacological deficiencies, i.e., poor proteolytic and high immunogenic stabilities, severely limiting their clinical application. It is very necessary to adjust the mechanical properties and degradation time of these chiral nanomaterials according to needs. The combined preparation of supramolecular complexes and polymers is an effective strategy to achieve enhanced stability, so its structure can be optimized and then reassembled to promote stability. Additionally, whether the use of high-power lasers for the stimulation of tumor cell death presents the risk of long-term radiation damage to healthy cells also is another critical issue. Therefore, the application of biomarkers to chiral nanomaterials may provide a clear boundary for near-infrared light to irradiate tumor tissues, thereby minimizing the harmful effects of light on normal tissues.

Although this nanomaterials-based anti-tumor strategy is still at the laboratory stage of development and presents many challenges to be overcome, we believe that chiral nanomaterials show enormous promise and the potential to become a crucial tool in precision medicine.

## Data Availability

Without restrictions.
